# Nasopharyngeal Cancer: Prevalence, Outcome, and Impact on Health-Related Quality of Life at Princess Norah Oncology Center, Jeddah, Saudi Arabia

**DOI:** 10.7759/cureus.8199

**Published:** 2020-05-19

**Authors:** Nabil Alsafadi, Mohammed S Alqarni, Meshari Attar, Rayan Mgarry, Abdulhameed Bokhari

**Affiliations:** 1 Radiation Oncology, King Abdulaziz Medical City, National Guard Hospital Jeddah, Jeddah, SAU; 2 Medicine, King Saud Bin Abdulaziz University for Health Sciences, Jeddah, SAU; 3 Urology, King Saud Bin Abdulaziz University for Health Sciences / International Medical Center, Jeddah, SAU

**Keywords:** nasopharyngeal carcinoma, outcomes, heath related quality of life, prevalence, kingdom of saudi arabia (ksa)

## Abstract

Introduction

Head and neck malignancies are considered among the most common cancers that arise from different anatomical sites in the region. The number of new cases diagnosed worldwide each year is estimated to be more than 550,000 resulting in about 380,000 deaths. One of these head and neck cancers that may affect patient quality of life is the nasopharyngeal carcinoma (NPC). The purpose of our study is to assess the outcome, and the quality of life of these patients. Our study reviews NPC patients treated at Princess Norah Oncology Center, Jeddah, retrospectively over the past 15 years to provide additional information on this disease in Saudi Arabia.

Methods

We included all histologically confirmed cases of NPC seen at National Guard Hospital in Jeddah between 2002 and 2017. The data was collected retrospectively from the BestCare system, hospital information system, and the medical records. The created table included demographics, comorbidities, and first symptoms. The research table also contained stage at presentation and treatment modalities. Moreover, 25 patients were asked to complete the Arabic versions of European Organisation for Research and Treatment of Cancer Quality of Life Questionnaire Head and Neck Module (EORTC HN-35) module questionnaire to assess the quality of life. All results were computed using IBM SPSS version 23 (IBM Corp., Armonk, NY), which was provided by College of Medicine at KSAU.

Results

A total of 107 patients with adequate documentation were identified. There were 72.9% males and 27.1% females; 81.3% of patients were alive and in remission and 18.7% were dead. Neck mass was the most common clinical manifestation present in 84.1% of patients. Radiotherapy and chemotherapy were the most used modality by 96.3%. The five-year survival rate year was 81.3%. Moreover, the H&N-35 questionnaire showed that the NPC survivors suffered mostly poor social contact.

Conclusion

A large proportion of the identified patients were in remission. Quality of life assessment shows that the main impact of the disease and treatment was on social contact.

## Introduction

According to the World Health Organization (WHO), cancer is one of the leading causes of mortality and morbidity globally [1]. Head and neck malignancies are among the most common cancers that arise from different anatomical sites in the region. The number of new cases diagnosed worldwide each year is estimated to be more than 550,000 cases resulting in about 380,000 deaths [2,3].

A specific type of cancer that occurs between the head and neck region is nasopharyngeal cancer (NPC), which is very rare in the West [4]. Worldwide, the incidence of NPC is slightly less than one per 100,000 population annually [5]. In Saudi Arabia, NPC is seen in 6% of all malignancies that are diagnosed yearly with an incidence of 0.8 per 100,000 [6]. NPC is the most common type of malignancy arising in the nasopharynx, the narrow tubular passage behind the nasal cavity [7,8]. It is characterized often by poorly or undifferentiated carcinoma. It differs from non-nasopharyngeal head and neck squamous cell carcinomas in several ways, including its association with the Epstein-Barr virus (EBV), increased radio-chemo sensitivity, and a greater propensity for distant metastases [9]. The risk to develop NPC seems related to several factors, such as EBV latent infection, environmental factors, which includes the high intake of preserved foods along with smoking, and genetic predisposition [10,11]. In NPC viral DNA infects epithelial cells and is associated with their transformation to cancer.

The genetic factor seems to impact the development of the NPC dramatically [12,13]. There is a high rate of this cancer within some specific ethnic groups. Patients that have A2 HLA haplotypes, and cytogenetic abnormalities identified within the tumor samples [14]. Clinical presentations related to NPC may not appear in early stages. However, in late stages, NPC includes apparent complications such as swollen lymph nodes around the neck, blood in the nose and saliva, hearing loss and ear infection [14,15]. Moreover, advanced NPC can lead to a multiple of neurological associated symptoms, including unilateral deafness and difficulty in opening the mouth, and this is known as Trotter’s syndrome [16]. Serious complications of nasopharyngeal carcinoma can also occur during treatment. According to a review article by Suarez et al., radiotherapy and radio-surgery for NPC can include some complications such as nasopharyngeal necrosis, temporal lobe necrosis, cranial nerve palsies and cerebral edema mainly in patients retreated for local recurrence [17]. In addition to that nasopharyngeal cancer can adversely affect quality of life, including emotional, social and psychological abnormalities [18]. NPC patients can also be presented with difficulty swallowing, hearing loss, and speech difficulties [19]. Therefore, it is important to assess quality of life in these patients.

The purpose of our study is to find the morbidity, functional status and to assess the quality of life of these patients. Our study reviews NPC patients treated at Princess Norah Oncology Center, Jeddah, retrospectively over the past 15 years to provide additional information on nasopharyngeal cancer patients in Saudi Arabia.

## Materials and methods

The records of 141 clinical data were collected from all histologically confirmed cases of NPC which took place between 2002 and 2017, at National Guard Hospital in Jeddah, Kingdom of Saudi Arabia. The inclusion and exclusion criteria were retrospectively reviewed on hard files and soft files (BestCare). Patients that had been diagnosed before 2002 or had other nasopharyngeal malignancies were excluded. The research started by gathering 141 patients’ files, and 34 NPC patients were excluded because their files were either not complete or were lost follow-up, so we included 107 NPC patients in the incidence of the study.

A table was created that included the following demographics: age, gender, remission status and death status. Headache, hearing loss, and vision disturbances were the symptoms included in the table. The research table also contained stage of cancer at presentation, tumor nodes metastasis (TNM) stages, radiation, chemotherapy, surgery, hypertension and diabetes. Staging of the disease was stated according to the Cancer Staging Manual by the American Joint Committee on Cancer, sixth edition [20]. All patients’ information was viewed and then placed into the table accordingly. The treatment modalities were radiotherapy, chemotherapy and surgery.

Moreover, 25 patients, who attended the follow-up clinic during the study period and were free of the disease more than two years, were asked to complete Arabic versions of European Organisation for Research and Treatment of Cancer Quality of Life Questionnaire Head and Neck Module (EORTC HN-35) questionnaire to assess the quality of life related to the stage and treatment by the research coordinator. Sub-scales have measured pain, swallowing, sense, speech, social eating and contact, weight loss/gain, painkillers, ill feeling, coughing, feeding tube, open and dry mouth, teeth, sticky saliva and sexual problems. For the analysis, categorical variables were summarized by frequencies and percentages, and continuous variables by means and standard deviations, or medians and interquartile ranges if their distributions were skewed. Variables with p value less than .05 were considered significant. All results were computed using IBM SPSS version 23 (IBM Corp., Armonk, NY), which was provided by College of Medicine at KSAU.

## Results

**Figure 1:**
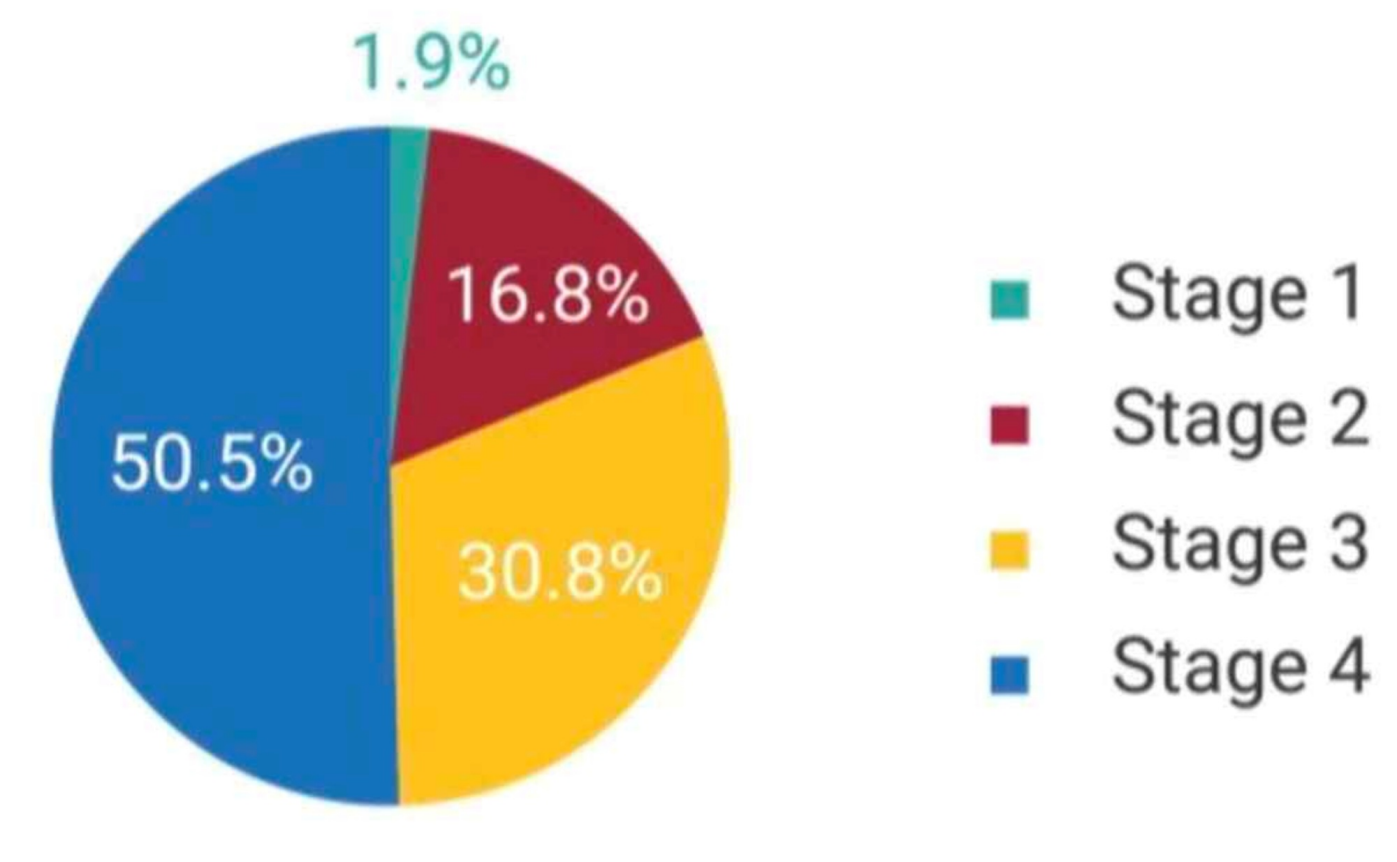
Patients’ stages

Our sample size included 107 patients. Patients in this study were ranged from 11 to 106 years with a mean age of 53.85 years (SD = 15.72 years), had a BMI ranged from 13.42 to 51.20 with a mean BMI of (SD = 7.06) and the mean follow-up was 84.42 months (SD = 55.78). Among our population, 72.9% (n = 78) were males and 27.1% (n = 29) were females. They had co-morbidities such as diabetes (25.2%, n = 27), hypertension (29%, n = 31), and smoking (9.3%, n = 10); 81.3% (n = 87) of our population presented with remission and 18.7% (n = 20) patients were dead. The patients’ stages and clinical presentations are shown in Figure 1 and Tables 1, 2.

**Table 1:** Clinical presentations

Clinical presentation	N (%)
Headache	45 (42.1%)
Nasal obstruction	38 (35.5%)
Nasal bleeding	32 (29.9%)
Visual symptoms	33 (30.8%)
Decreased hearing	47 (43.9%)
Neck mass	90 (84.1%)
Cranial III involvement	3 (2.8%)
Cranial V involvement	1 (0.9%)
Cranial VI involvement	6 (5.6%)
Cranial VII involvement	5 (4.7%)

**Table 2:** TNM classifications TNM: Tumor Nodes Metastasis

TNM Classifications	N (%)
T classifications	
T1	9 (8.4%)
T2	23 (21.5%)
T3	35 (32.7%)
T4	40 (37.4%)
N classifications	
N0	10 (9.3%)
N1	20 (18.7%)
N2	45 (42.1%)
N3	32 (29.9%)
M classifications	
M0	90 (84.1%)
M1	17 (15.9%)
Pathology	N (%)
Squamous cell carcinoma	16 (15%)
Undifferentiated carcinoma	82 (76.6%)
Differentiated was not stated	9 (8.4%)

**Figure 2:**
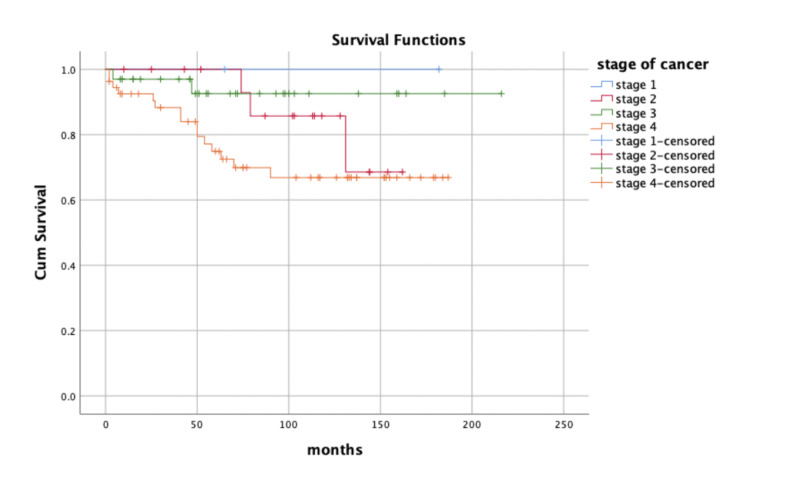
Overall five-year survival by stages

The overall five-year survival rate was 81.3% for the whole group (Figure 2) and was 100% for stage 1 and 83.3% for stage 2. The five-year survival rate was 93.9% and 72.2% for those with stage 3 and 4, respectively. Patients with stage 1 and 2 have higher five-year survival but statistically insignificant (p-value > 0.05).

96.3% patients (n = 103) completed the treatment with radiotherapy and/or chemotherapy, and 20.6% patients (n = 22) underwent any type of surgery. The assessment of quality of life (QoL) of 25 patients, who were in remission, was asked by research coordinator using H&N-35 modules. A calculated score for each patient is shown in Table 3. In the H&N-35 module, swallowing and social contact ranked the highest scores.

**Table 3:** Calculated scores for QLQ-H&N-35

Symptoms	N	Minimum	Maximum	Mean	Std. Deviation
Pain	25	4.00	12.00	5.4000	2.06155
Swallowing	25	4.00	12.00	7.0400	2.57358
Sense Problems	25	2.00	6.00	3.2400	1.12842
Speech Problems	25	3.00	9.00	4.5600	1.93821
Social Eating	25	4.00	16.00	6.7200	3.22128
Social Contact	25	5.00	18.00	7.3200	3.09192
Less Sexuality	25	2.00	10.00	4.2400	2.94788
Teeth	25	1.00	4.00	1.8800	0.88129
Opening Mouth	25	1.00	4.00	2.0400	1.17189
Dry Mouth	25	1.00	4.00	2.1200	1.05357
Sticky Saliva	25	1.00	4.00	2.2800	0.89069
Coughing	25	1.00	3.00	1.7200	0.73711
Feel of Illness	25	1.00	4.00	1.6400	0.86023
Pain Killers	25	6.00	7.00	6.5200	0.50990
Nutritional Supplements	25	6.00	7.00	6.5200	0.50990
Feeding Tube	25	6.00	7.00	6.9200	0.27689
Weight Loss	25	6.00	7.00	6.8000	0.40825
Weight Gain	25	6.00	7.00	6.2400	0.43589

## Discussion

Patients presenting with early stage disease had a higher five-year survival rate though not statistically significant, compared to more advanced stage, with stage 1 being 100%. Similarly, a study that was done in China concluded that stage 1 NPC patients had 100% five-year survival rate [21]. In practice, the commonest presentation in our series was neck nodes, denoting more advanced stage. Furthermore, the incidence rate of our NPC patients was higher in males compared to females with a male-to-female ratio of almost 3:1, similar to the results from the International Agency for Research on Cancer at WHO (WHO-IARC) that is seen in NPC patients in Kuwait [22]. The association between mortality rate and patients’ risk factors such as diabetes, hypertension, and smoking was statistically insignificant in our study.

From the quality of life results, the social aspect was the most affected, as patients reported that they had issues with their social life. 72% of the surveyed patients had difficulty staying in touch with relatives, 64% reported having problems while eating a meal with others and 36% stated having speech difficulties. These multiple social difficulties may present in patients with various cancer types, however, we believe that the social aspect is crucial in NPC patients because of the younger age, and higher cure rate.

In addition, only 12% of the patients in our study reported a feeling of illness, and 16% reported they need to use any type of painkillers. Also, 16% reported having mouth dryness, sticky saliva and cough. Though not related to the social aspect, these may suggest better quality of life in the NPC patients day-to-day living considering that they have cancer. In comparison, a study done in Turkey showed swallowing, mouth, teeth problems and weight gain were higher in their population but statistically insignificant [23].

A significant number of our patients were excluded due mainly to incomplete clinical data and/or follow-up, making interpretation of the survival data limited. This is in agreement with another study from Saudi Arabia, where differences in survival rates lacked statistical significance due to the low number of patients and a loss of follow-ups [24].

## Conclusions

This study included 15-year retrospective data about nasopharyngeal cancer patients in Princess Norah Center for Cancer, and we were able to identify 107 NPC patients with adequate information. Higher incidence rate was in male patients, and the neck mass was the most common clinical presentation that accounted for 84.1%. In addition, there was no association between mortality rate and patients’ co-morbid factors. Also, social contact among our patients was a major issue affecting the quality of life. Future studies must focus on ways to improve the functional outcome of this young patient population.
